# Comprehensive next-generation sequencing reveals low-grade fibromyxoid sarcoma of the vulva missed by morphological diagnosis: a case report

**DOI:** 10.3389/fmed.2023.1343407

**Published:** 2024-01-16

**Authors:** Shuang Tan, Hongruo Liu, Evenki Pan, Siye Liu, Jiangyan Zhang, Jie Wang, Ning Wang

**Affiliations:** ^1^Department of Gynecology, The Second Affiliated Hospital of Dalian Medical University, Dalian, China; ^2^Department of Medical Oncology, The Second Affiliated Hospital of Dalian Medical University, Dalian, China; ^3^Department of Medical, Nanjing Geneseeq Technology Inc., Nanjing, Jiangsu, China; ^4^Department of Pathology, The Second Affiliated Hospital of Dalian Medical University, Dalian, China

**Keywords:** low-grade fibromyxoid sarcoma, vulva, MUC4 protein, *FUS-CREB3L2* fusion, next-generation sequencing

## Abstract

Low-grade fibromyxoid sarcoma (LGFMS) is a rare soft tissue tumor composed of bland spindled cells in a variably fibrous to myxoid stroma. Its occurrence in the vulva region is rare, and thus, it may not be always taken into account in the differential diagnosis. Here, we describe a 34-year-old woman presented with a right vulvar mass and underwent complete surgical excision. The final pathologic diagnosis revealed LGFMS of the vulva based on the morphological, immunophenotypic, and molecular genetic features. The patient has not experienced a local or metastatic recurrence after 9-month follow-up. Despite being rare, LGFMS of the vulva should be considered when making a diagnosis of vulvar lesions. We also report that the genetic testing by next-generation sequencing (NGS) represents a very useful tool for the differential diagnosis of LGFMS from its mimics. Moreover, we have reviewed the literature on LGFMS of the vulva and summarized the characteristics of the patients, providing assistance for the diagnosis of such patients. Most vulvovaginal LGFMS can be fully removed through surgery. However, ongoing monitoring over the long term is essential as local and/or distant spread can occur decades after the initial diagnosis.

## Introduction

Low-grade fibromyxoid sarcoma (LGFMS) is a rare sarcoma subtype, which mainly occurs in younger adults with equal predilection for men and women (1). Sarcomas account for 1% of adult cancer, while LGFMS is estimated to represent less than 5% of soft tissue sarcomas (2). LGFMS typically presents in the trunk and limbs and is composed of bland spindle cells in a variably fibrous to myxoid stroma. This tumor is similar to other sarcomas. The overexpression of MUC4 can be used as a specific immunohistological marker of LGFMS and SEF. The fusion of *FUS-CREB3L2* caused by t(7;16)(q32-34;p11) chromosomal translocation is the most frequently seen (75–95% of patients with LGFMS) (3). Other rare fusions are *FUS-CREB3L1* and *EWSR1-CREB3L1* (4). NGS has been applied to solve the problem of identifying cancer subtype. LGFMS is rarely described in the vulva, and limited literature has been published yet. In this report, we describe a rare case of LGFMS that occurred in the vulvar., which showed bland spindle cells in a variably fibrous to myxoid stroma. Additionally, the tumor exhibited positive MUC4 expression. Further molecular analysis using targeted next-generation sequencing (NGS) identified *FUS-CREB3L2* gene fusion. This finding was subsequently confirmed by fluorescence *in situ* hybridization (FISH). LGFMS is a tumor with low-grade histological features but carries a high risk of local recurrence and a considerable risk of metastasis. Thus, it is important to identify and manage these patients at an early stage. To better characterize this tumor and raise awareness about its occurrence in the vulva, this report also presents a review of the literature on LGFMS of the vulva and discusses the differential diagnosis.

## Case presentation

**Figure 1:**
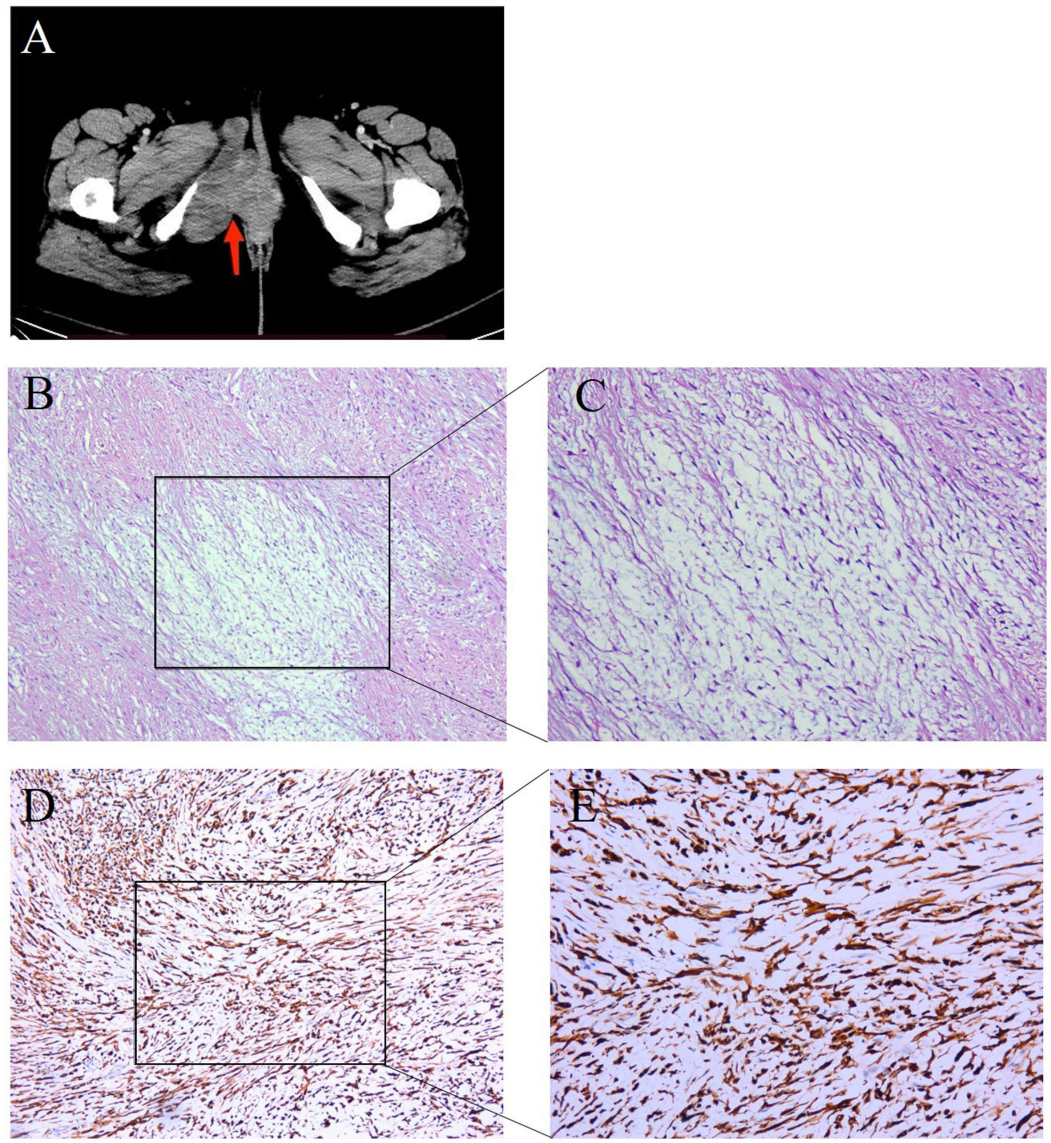
Representative clinical images for the tumor tissue in the right vulvar. **(A)** Computed tomography (CT) scans showed the patient’s tumor mass (arrows). **(B,C)** HE staining (100× and 200×) of the tumor tissue showed bland spindle cells in a variably fibrous to myxoid stroma. In the fibrous area, the tumor cells showed a swirling growth or short bundle pattern that mixed with collagen fibers, whereas myxoid foci contained arched-shaped capillaries. **(D,E)** IHC examinations (100× and 200×) of the tumor tissue were positive for MUC4.

A 34-year-old woman was referred for further investigation and treatment of a mass in the vulvar due to intermittent pain for 1 year. There was no associated pain or paresthesia. In 2007, she was initially diagnosed with benign neurofibroma, which was removed by complete resection. She had no family cancer history. In July 2022, a computed tomography (CT) revealed that the tumor recurred and had significantly increased to 10 × 3 × 3 cm (Figure 1A). This recurrent mass was close to the descending branch of the pubic bone and urethra. No abnormalities were found in the vagina, cervix, uterus, and bilateral adnexal regions. The surgical resection was performed. The pathology shows that the tumor was composed of bland spindle cells in a variably fibrous to myxoid stroma using hematoxylin and eosin staining (Supplementary methods). In fibrous areas, the tumor cells showed a swirling growth or short bundle pattern that mixed with collagen fibers. In the myxoid areas, the capillaries formed arch-like structures. The cell boundary was obscure. There was no necrosis present and no areas characteristic of sclerosing epithelioid fibrosarcoma (SEF) (Figures 1B,C). Immunohistochemistry (IHC) analysis showed positive staining for high-mobility group AT-hook 2 (HMGA2), signal transducer and activator of transcription 6 (STAT6), Rb, and β-catenin and negative staining for ER, smooth muscle myosin heavy chain (SMMHC), ALK, phosphorylated histone H3 (PHH3), CD34, PR, S-100, SOX10, caldesmon, and SMA (Supplementary Figure S1). The Ki67 index was 2% (Supplementary Figure S1).

**Figure 2:**
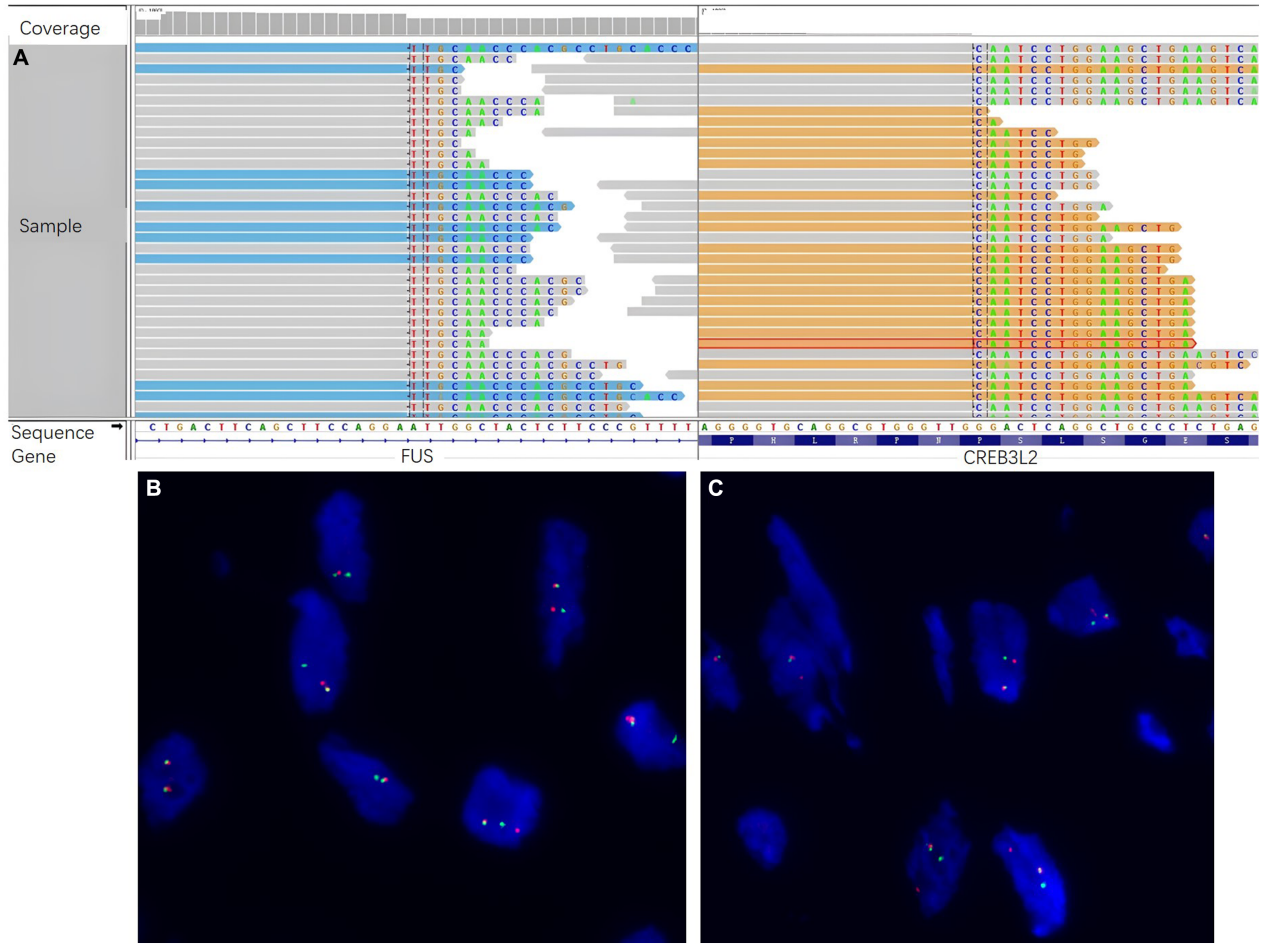
Molecular genetic studies by next-generation sequencing (NGS) and fluorescence *in situ* hybridization (FISH). **(A)** NGS sequencing reads indicating a fusion gene between *FUS* exon 6 and *CREB3L2* exon 5 were demonstrated by Integrative Genomics Viewer (IGV) software. FISH analyses demonstrated positive for rearrangements of both the **(B)** *FUS* and **(C)** *CREB3L2* loci, using the break-apart probe sets.

It was still difficult to diagnose the type of tumor, an LGFMS or an aggressive angiomyxoma (AAM), a distinctive neoplasm occurring in the female perineum and pelvis. There were many IHC markers for sarcoma; however, to conserve tissue samples, we conducted genetic testing using targeted NGS in November 2022. The analysis focused on 481 soft tissue and bone tumor-related genes (Nanjing Geneseeq Technology Inc.) (Supplementary methods), which revealed a fusion gene between *FUS* exon 6 and *CREB3L2* exon 5 with a variant allele frequency (VAF) of 43.31% (Figure 2A). In addition, all the somatic mutations are presented in Supplementary Table S1. FISH analyses using the break-apart probe sets demonstrated positive rearrangements of both the *FUS* and *CREB3L2* loci, thus confirming the NGS results (Figures 2B,C). Additionally, the IHC of MUC4 was done, and its positivity supported the diagnosis of an LGFMS (Figures 1D,E). Computed tomography (CT) scan showed no distant metastasis. On the basis of the patient’s age, morphological, immunophenotypic, and molecular genetic features, a diagnosis of LGFMS was determined. The tumor was removed with clear margins. After her most recent clinical follow-up at 12 months post-surgery, the status of the patient remained stable without local or distant recurrence.

## Discussion

LGFMS is a rare fibrosarcoma, which is mainly found in deep soft tissues of the proximal extremities or trunk of young adults. Its occurrence in the female vulva is extremely rare, and thus, it may not be first considered in the differential diagnosis. To the best of our knowledge, there have been 10 cases of LGFMS that occurred in the vulva: four individual case reports (5–8) and a study cohort of seven cases of which six were in the vulva (9) (Table 1). The mean age at presentation was 40 years ranging between 22 and 59 years. The tumor size ranged from 2 to more than 10.2 cm. In our case, the patient presented with a 10 × 3 × 3 cm mass in the right labia majora. Similarly, most patients have a mass on the labia majora, which is less frequent on the labia minora, perineum, clitoris, and mons (8). Recognition of vulvovaginal LGFMS is complicated due to its rarity at this site. Consequently, a broad differential diagnosis must be considered, such as SEF, AAM, perineurioma, myxoid smooth muscle neoplasia, cellular angiofibroma, and myxoid dermatofibrosarcoma protuberans.

**Table 1:** Literature review of low-grade fibromyxoid sarcoma of the vulva.

Author, year	Ethnicity	Number of cases	Age at diagnosis	Tumor size (cm)	MUC4	*FUS* rearrangement	Treatment	Survival
Barnhill et al., 2012 (6)	Caucasian	1	45	5	NA	NA	Left radical hemivulvectomy + ipsilateral inguinal lymph-node resections	RFS:12 mos
Van Sandt et al., 2013 (5)	Caucasian	1	36	6	+	+	Right hemivulvectomy	RFS:24 mos
Cengiz et al., 2018 (8)	Caucasian	1	45	2	+	NA	Left radical hemivulvectomy	RFS:24 mos
Costigan et al., 2022 (9)	Caucasian	6	59,37,40.46,39,34	2,5, 8.7, 1 0.2 (1 NA)	+ (5/6)	+ (5/7)	5 surgical excision (1 NA)	RFS:141,16,57 and 10 mos (2 NA)
Sahraoui et al., 2022 (7)	Caucasian	1	22	3	+	NA	Radical hemivulvectomy	RFS:24 mos
Current case, 2023	Mongolian	1	34	10	+	+	Surgical excision	RFS:7 mos

OS, Overall survival; RFS, Relapse-free survival; NA, Not available; mos, Months.

Histologically, LGFMS was characterized by bland-appearing fibroblastic spindle cells and similar to other soft tissue sarcoma. In our case, the tumor cells showed a swirling growth or short bundle pattern that mixed with collagen fibers in the fibrous area, whereas myxoid foci contained arched-shaped capillaries. There were no areas characteristic of SEF. The patient was first diagnosed with AAM on the basis of morphology. However, due to the uncertainty of the proposed diagnosis, the identification of molecular characteristics via NGS was performed. A *FUS-CREB3L2* fusion was revealed at a VAF of 43.31%. Over 90% of LGFMS patients have a pathognomonic *FUS-CREB3L2* fusion, with a minority harboring *FUS-CREB3L1* or *EWSR1-CREB3L1* fusions (10–12). Costigan et al. also reported a series of seven cases of LGFMS in the lower female genital tract, five of which harbored a *FUS* rearrangement (9). Therefore, genetic testing by NGS represents a very useful tool for the differential diagnosis of LGFMS from its mimics. Additionally, MUC4 is currently considered to be a highly sensitive and specific indicator of LGFMS because it is only positive in LGFMS and a small subset of monophasic synovial sarcomas (13). In the current case, the diagnosis of LGFMS was determined with MUC4 expressed in tumor cells. Since the molecular characteristics via NGS helped to narrow the field of the differential diagnosis, MUC4 immunohistochemistry is a verification method to avoid wasting tumor samples for other immunohistochemical markers of sarcoma. Due to the rarity of LGFMS at gynecological locations and the overlap with more common morphological mimics, it is crucial to maintain a high level of alertness to avoid misdiagnosis. Positive MUC4 immunostaining, along with molecular testing for *FUS* (or, less commonly, *EWSR1*) rearrangement, facilitates the early diagnosis of LGFMS. This enables the implementation of personalized treatment strategies, which contribute to an optimal recovery.

Complete surgical resection with clear margins is the standard treatment for LGFMS. In a case series of 36 patients with LGFMS treated with surgical resection, 5- and 15-year local control rates were 83 and 79%, respectively (14). The prognosis of patients with LGFMS in the vulva after complete surgical resection or radical hemivulvectomy seems good without developing recurrence after a 2-year follow-up. Given the rarity of LGFMS in the vulva, we also investigated the treatment strategies for other sarcomas in the lower female genital tract. Wide surgical excision is the gold-standard treatment for AAM. However, AAM typically has a high recurrence rate of between 36 and 72% after surgery (15). Dierickx et al. recommended that incomplete surgical excision may be an option for AA of the vulva, but the effectiveness of hormonal treatment and radiation therapy is not clear yet (16). Uterine smooth muscle tumor of uncertain malignant potential (STUMP) represents a group of neoplasms that originate from the smooth muscle cells within the uterine wall. The primary treatment for STUMP is total hysterectomy with or without bilateral adnexectomy, whereas myomectomy alone can be considered in patients who desire to preserve their fertility (17, 18). In our case, the patient was stable 7 months following surgical resection. Due to its indolent clinical behavior, LGFMS is considered to be insensitive to radiotherapy and chemotherapy (4). The development of targeted therapy and immunotherapy has remained an ongoing challenge for LGFMS. In this setting, the identification of molecular characteristics via NGS is a promising avenue for future therapeutic approaches.

The limitation of the single-case presentation in this study should be noted. Therefore, it is essential to further evaluate the characteristics of vulvar LGFMS within a larger cohort to reduce the likelihood of misdiagnosis. The lack of long-term surveillance is also a limitation of our case. We are unable to predict whether the patient may experience a recurrence or metastasis with longer follow-up periods.

## Conclusion

In summary, we herein report a rare LGFMS in the vulva. Due to its rarity in the lower female genital tract and the overlap with other common mimics, LGFMS can be misdiagnosed in the differential diagnosis. Correct diagnosis is crucial because of the high risk of recurrence and the possibility of late metastatic spread. In the proper morphological context, positive MUC4 immunostaining and molecular testing for *FUS* fusion are confirmatory. Especially molecular detection can save samples and avoid excessive IHC index testing. Complete surgical excision and long-term follow-up of the patient are required as local and/or distant spread can occur decades after the initial diagnosis. This study provides support for the characteristic research of LGFMS at gynecologic sites, offering evidence for the diagnosis and treatment of such cancers in the future.

## Data availability statement

The original contributions presented in the study are included in the article/Supplementary material, further inquiries can be directed to the corresponding authors.

## Ethics statement

Written informed consent was obtained from the individual(s) for the publication of any potentially identifiable images or data included in this article.

## Author contributions

ST: Methodology, Writing – original draft. HL: Investigation, Writing – original draft. EP: Writing – original draft. SL: Writing – original draft. JZ: Writing – review & editing. JW: Writing – review & editing. NW: Conceptualization, Writing – review & editing.
