# Supplementary material for: Comprehensive next-generation sequencing reveals low-grade fibromyxoid sarcoma of the vulva missed by morphological diagnosis: a case report
Source: Front Med (Lausanne). 2024 Jan 16;10:1343407. doi: 10.3389/fmed.2023.1343407 (PMC10824949; doi:10.3389/fmed.2023.1343407)
Supplement: Supplementary file 1 [file Data_Sheet_1.docx]

**Supplementary Methods**

*Hematoxylin and eosin staining*

Slice the tissue samples and submerge them in 10% neutral buffered formalin. The fixation should take place at a temperature of 25℃ for a duration of 3-6 hours. After fixation, dehydrate the tissue samples, embed them in paraffin, and prepare tissue sections that are 4 microns thick. Immerse the paraffin sections in xylene for 10 minutes, change the xylene and continue soaking for another 10 minutes to dissolve the wax. Decaxify using a gradient of ethanol concentrations (anhydrous ethanol, 95%, 85%, 70% ethanol), each immersion lasting 5 minutes. Clean the hydrated tissue sections by immersing them in PBS solution, each immersion lasting 5 minutes, repeated three times. Subsequently, stain them in hematoxylin for 10 minutes. Afterwards, rinse off the excess hematoxylin stain with distilled water. Differentiate the samples using 1% hydrochloric acid in ethanol, and rinse the sections thoroughly with distilled water. Complete the bluing process using 0.6% ammonia water, rinse with clean water, and then rinse the sections thoroughly with distilled water. Immerse the sections in eosin dye for 1 minute. Rinse the sections thoroughly with distilled water, then dehydrate the sections using a gradient of 80% ethanol for 5 seconds, 95% ethanol for 2 minutes, and anhydrous ethanol for 2 minutes. Immerse the dehydrated tissue sections in xylene twice, each immersion lasting 4 minutes. Finally, dry the tissue sections and seal them with neutral resin.

DNA extraction and targeted enrichment

FFPE genomic DNA was purified using the QIAamp DNA FFPE Tissue Kit (Qiagen). The DNA was quantified using the dsDNA HS Assay Kit on a Qubit Fluorometer (Life Technologies). Sequencing libraries were prepared using the KAPA Hyper Prep Kit (KAPA Biosystems), as described previously (1). Indexed DNA libraries were pooled together for probe-based hybridization capture of the targeted gene regions covering 481 soft tissue and bone tumors related genes.

*Sequencing data processing*

Sequencing was performed using the Illumina HiSeq4000 platform, followed by data analysis as previously described (2). In brief, sequencing data were analyzed by Trimmomatic (3) to remove low-quality (quality <15) or N bases, and were then mapped to the human reference genome, hg19, using the Burrows-Wheeler Aligner (https://github.com/lh3/bwa/tree/master/bwakit). PCR duplicates were removed by Picard (available at https://broadinstitute.github.io/picard/). The Genome Analysis Toolkit (GATK) (https://software.broadinstitute.org/gatk/) was used to perform local realignments around indels and base quality reassurance. Gene fusions were identified by FACTERA (4). Somatic SNPs and indels were analyzed by VarScan2 (5) and Mutect2, with the mutant allele frequency cutoff at 2% for tissue samples and a minimum of three unique mutant reads. Common SNPs were excluded if they were present in >1% population frequency in the 1000 Genomes Project or the Exome Aggregation Consortium (ExAC) 65,000 exomes database. The resulting mutation list was further filtered by an in-house list of recurrent artifacts based on a normal pool of whole blood samples.

1. Y. Shu, X. Wu, X. Tong, X. Wang, Z. Chang, Y. Mao, X. Chen, J. Sun, Z. Wang, Z. Hong, L. Zhu, C. Zhu, J. Chen, Y. Liang, H. Shao, and Y.W. Shao. (2017). Circulating Tumor DNA Mutation Profiling by Targeted Next Generation Sequencing Provides Guidance for Personalized Treatments in Multiple Cancer Types. Sci Rep 7, 583.

2. Z. Yang, N. Yang, Q. Ou, Y. Xiang, T. Jiang, X. Wu, H. Bao, X. Tong, X. Wang, Y.W. Shao, Y. Liu, Y. Wang, and C. Zhou. (2018). Investigating Novel Resistance Mechanisms to Third-Generation EGFR Tyrosine Kinase Inhibitor Osimertinib in Non-Small Cell Lung Cancer Patients. Clin Cancer Res 24, 3097-3107.

3. A.M. Bolger, M. Lohse, and B. Usadel. (2014). Trimmomatic: a flexible trimmer for Illumina sequence data. Bioinformatics 30, 2114-20.

4. A.M. Newman, S.V. Bratman, H. Stehr, L.J. Lee, C.L. Liu, M. Diehn, and A.A. Alizadeh. (2014). FACTERA: a practical method for the discovery of genomic rearrangements at breakpoint resolution. Bioinformatics 30, 3390-3.

5. D.C. Koboldt, Q. Zhang, D.E. Larson, D. Shen, M.D. McLellan, L. Lin, C.A. Miller, E.R. Mardis, L. Ding, and R.K. Wilson. (2012). VarScan 2: somatic mutation and copy number alteration discovery in cancer by exome sequencing. Genome Res 22, 568-76.
