# Supplementary figures and images for: Comprehensive next-generation sequencing reveals low-grade fibromyxoid sarcoma of the vulva missed by morphological diagnosis: a case report
Source: Front Med (Lausanne). 2024 Jan 16;10:1343407. doi: 10.3389/fmed.2023.1343407 (PMC10824949; doi:10.3389/fmed.2023.1343407)

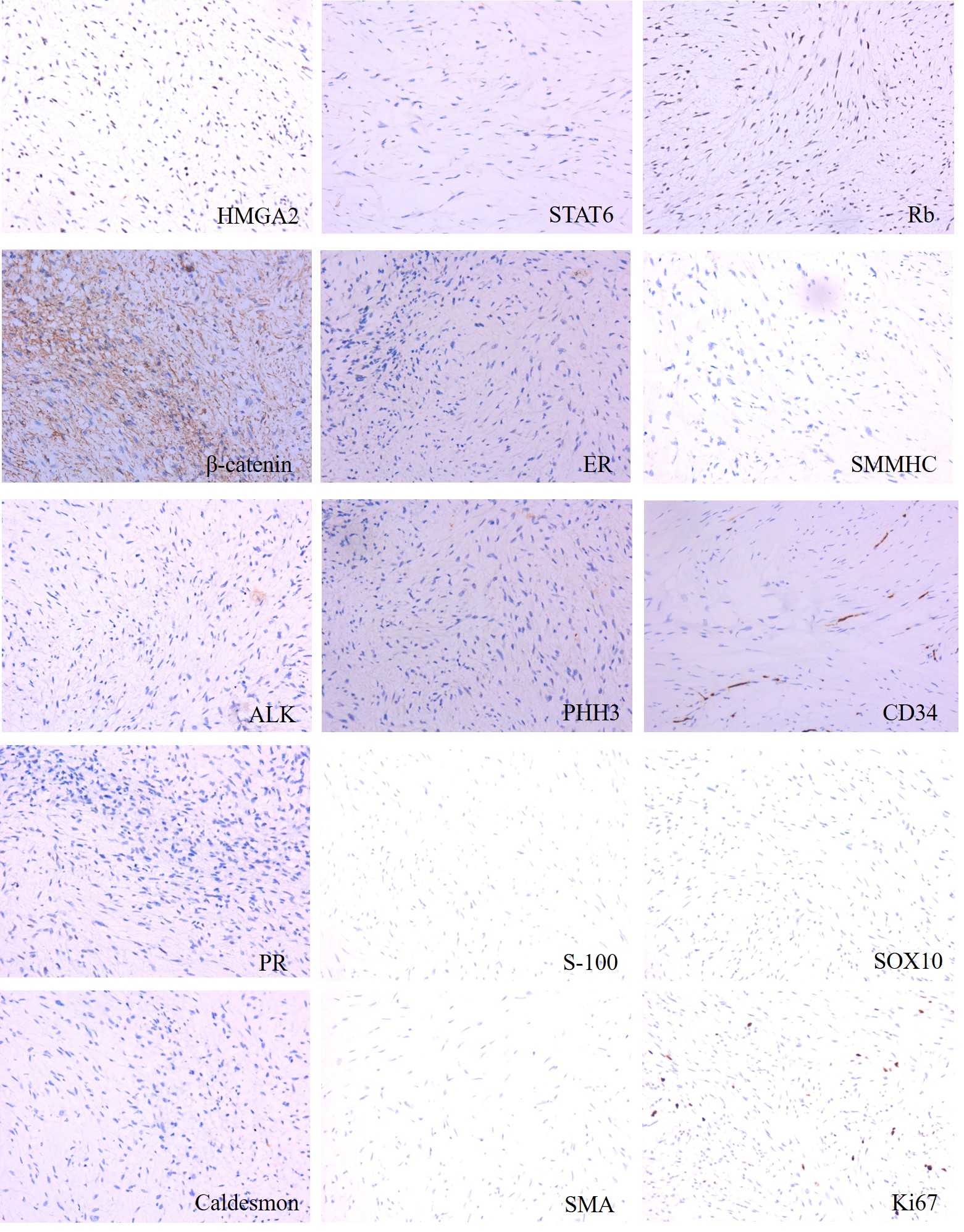

Supplement: Supplementary file 2 [file Image_1.JPEG]
